# Nanosecond pulsed electric fields promoting the proliferation of porcine iliac endothelial cells: An in vitro study

**DOI:** 10.1371/journal.pone.0196688

**Published:** 2018-05-01

**Authors:** Yuchen Zhang, Feihong Dong, Zhengxin Liu, Jinsong Guo, Jue Zhang, Jing Fang

**Affiliations:** 1 Department of Cardiology, Beijing Anzhen Hospital, Capital Medical University, Beijing, China; 2 Academy for Advanced Interdisciplinary Studies, Peking University, Beijing, China; 3 College of Engineering, Peking University, Beijing, China; Hungarian Academy of Sciences, HUNGARY

## Abstract

Currently, nanosecond pulsed electric fields (nsPEFs) with short pulse duration and non-thermal effects have various potential applications in medicine and biology, especially in tumor ablation. Additionally, there are a few investigations on its proliferative effects in the normal cell. Clinically, proliferation of endothelial cells can perhaps accelerate the stent endothelialization and reduce the risk of acute thrombosis. To explore the feasibility using nsPEFs to induce proliferation of endothelial cells, in this study, porcine iliac endothelial (PIEC) cell line was cultured and tested by CCK-8 assay after nsPEFs treatment. The results reflected that nsPEFs with low field strength (100ns, 5 kV/cm, 10 pulses) had a significant proliferative effect with an increase in the PIEC cell growth of 16% after a 48 hour’ post-treatment. To further understand the mechanism of cell proliferation, intracellular Ca^2+^ concentration was measured through fluo-4 AM and reactive oxygen species assay was applied to estimate the level of intracellular reactive oxygen species (ROS). Finally, the total nitric oxide assay for NO production in the cultured medium was evaluated. An enhanced concentration of intracellular Ca^2+^ and ROS were observed, while the concentration of extracellular NO also increased after nsPEFs treatment. Such experimental results demonstrated that nsPEFs with appropriate pulse parameters could effectively enhance cell proliferation on PIEC cells, and the cell proliferation associated strongly with the changes of intracellular Ca^2+^ concertation, ROS and NO production induced by nsPEFs treatment. This in vitro preliminary study indicates that as a novel physical doping, the nsPEFs have potential in stimulating endothelial cells to accelerate stent endothelialization.

## Introduction

Coronary artery stenosis is one of the major cardiovascular diseases, which cause myocardial infarction and peripheral artery disease worldwide [1]. Implanted stents are a widely used method for the treatment of such diseases [2]. While stent implantation expands the stenotic vessel and increases the lumen area, in-stent restenosis (ISR) is a major risk factor for coronary stent implantation and remains a major concern for patients who had bare-metal stents implanted. Compared with bare-metal stents, drug-eluting stents (DESs) is able to significantly reduce the risk of vascular restenosis and neointimal hyperplasia [3, 4]. However, drug-eluting stents improve vascular restenosis and also simultaneously inhibit the process of endothelialization, so the repair of damaged blood vessels is also delayed, leading to late thrombosis and some other complications [5]. Those treatments cannot completely eliminate ISR and the risk for late thrombosis. Therefore, rapid surface endothelialization of a coronary stent has important significance, which can provide a strategy for improving the condition of thrombosis and minimizing restenosis [6].

Different methods have been attempted to accelerate the endothelialization on the stent surface. Wu [7] et al. determined that vascular endothelial growth factor (VEGF) overexpression is able to promote endothelial cell proliferation and accelerate stent endothelialization. Additionally, Shirota [8] et al. fabricated an intravascular stent seeded through endothelial progenitor cells (EPCs) and tested it in vitro. The process of accelerating endothelialization with anti-CD34 antibodies (EPCs capable of capturing blood in patients) has also been reported [9]. However, those methods could not be used for clinical treatment, primarily due to the limited cell proliferation and adherence on the stent. Therefore, finding a new method is critical in promoting the proliferation of endothelial progenitor cells or in accelerating the endothelialization process.

Typically, the effects of pulsed electric fields on biological cells have been investigated since the late 1950s. More recently, the duration of the electric fields has been shortened to nanoseconds [10]. Nanosecond pulsed electric fields (nsPEFs) with short pulse duration, low energy density and non-thermal effects possess numerous practical applications in both medicine and biology including Tumor ablation [11], gene transfection [12] and wound healing [13]. Throughout the recent years however, we discovered an interesting phenomenon in which the proliferation effect could be induced by nsPEFs under relatively low electric field strength. Several types of research have reported that nsPEFs could improve the growth of and *Haloxylon ammodendron* seeds [14], enhance the proliferation and dedifferentiation of chondrocytes [15] while also increasing the avermectins production in *Streptomyces avermitilis* [16]. In the process of implanting stents, it is very important to accelerate the endothelialization on the stent surface. Previous studies [7, 17]suggested that some factors (VEGF, HGF) are able to rapidly stimulate the proliferation of endothelial cells, which can accelerate stent endothelialization, thus improving the condition of thrombosis and minimizing restenosis.

Base on those considerations, we attempted to use nanosecond pulse techniques to stimulate the growth of porcine iliac endothelial cells. In this experiment, nanosecond pulse devices were used to treat PIEC cell suspensions and the PIEC cell line was tested by CCK-8 assay for cell proliferation, intracellular Ca^2+^ concertation was measured using the fluorescence Ca^2+^ indicator fluo-4 AM, the Reactive Oxygen Species Assay Kit for intracellular reactive oxygen species level and Total Nitric Oxide Assay Kit for NO production in the cultured medium.

## Material and method

### 2.1 Cell line and cell culture

The cell line used in this study was PIEC (Institute of Biochemistry and Cell Biology, Chinese Academy of Sciences, P. R. China, No: GNO15), derived spontaneously from a porcine iliac artery endothelial cell culture. PIEC cells were cultured in RPMI-1640 medium, supplemented with 10% dialyzed fetal calf serum, 1% penicillin/streptomycin and 2mM L-glutamine (all purchased from Beyotime Institute of Biotechnology, Jiangsu, China). PIEC cells were seeded in culture plate. For this study, the cells were maintained in a humidified atmosphere at 37°C and 5% CO2 and were removed by trypsinization (Trypsin 0.25% and EDTA 0.02%; Sigma-Aldrich, Beijing, china) and washed with PBS (Sigma-Aldrich, Beijing, china).

### 2.2 The nanosecond pulsed electric field treatment

**Fig 1:**
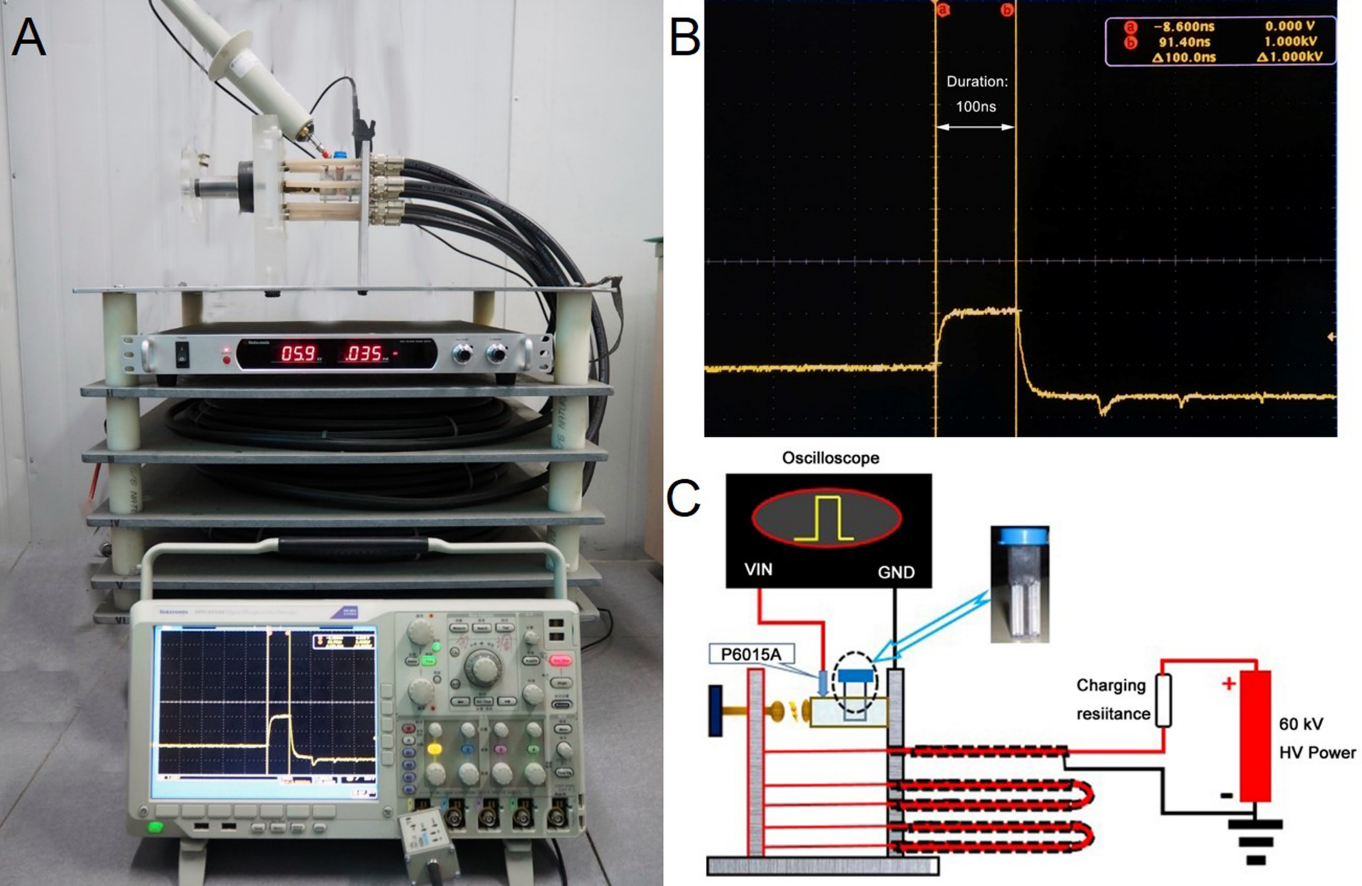
A schematic diagram of experimental setup for nsPEFs on PIEC cells. (A) The photo of 100 ns nsPEFs generator used in this experiment. (B) The typical waveforms of nsPEFs. (C) Circuit diagram of the basic Blumlein pulse forming system.

**Fig 2:**
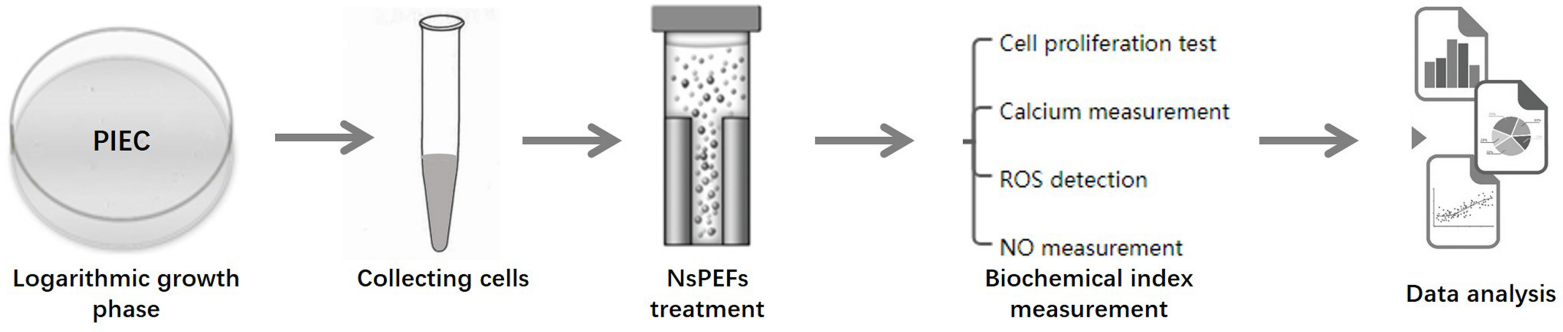
A schematic diagram of the experimental arrangement.

In this study, the pulsed high-voltage power supply and the nsPEF generator are showed in Fig 1. The nsPEF generator was applied as previously described [18]. The electric pulse exposed system consisted of a coaxial cable-based Blumlein pulse-forming network matched to the biological load in electroporation cuvette. PIEC cells were counted with a hemocytometer, and 1.1×10^6^ cells suspended in 1000 μL culture medium were added to 2mm gap cuvettes (Biosmith, aluminum plate electrode, San Diego, CA). The nsPEF generator produced nearly rectangular 100ns pulse that were delivered to the cells suspension by a 2 mm cuvette with 10 pulses at 5 kV/cm, 10 kV/cm and 20 kV/cm electric fields. Waveforms were monitored by using a digital phosphor oscilloscope (DPO4054. Tektronix. USA) equipped with a high voltage probe (P6015A. Tektronix. USA). A schematic diagram of the experimental arrangement is showed in Fig 2 and experiments were carried out under standard conditions except as noted. Samples were taken out of the cuvette for analyses at certain intervals. All experiments were conducted more than three times and the reproducibility was within 10%.

### 2.3 Cell proliferation test

In order to evaluate the effect of nsPEFs on PIEC cells, PIEC cells proliferation was investigated by Cell Counting Kit-8 (CCK-8) after incubation for 1 and 2 days respectively. When cells began to grow exponentially, they were treated with 10 pulses of nsPEFs with 100 ns durations at 5 kV/cm, 10 kV/cm, 20 kV/cm electric fields. In this study, we seeded 8.0×10^3^ cells/well into 96-well flat bottom (Corning Costar, Beijing, China) plates. After incubation for 24, 48h, 10 μl CCK-8 was added to each well, and cells were further incubated at 37°C for 2 h. The absorbance was measured at 450 nm by a microplate reader. All proliferation experiments were performed in triplicate. The cell viability (of control cells) and proliferation rate was calculate according to the following equation as usual.

The cell viability (of control cell) = (OD_nsPEFs_—OD_blank_)/(OD_control_—OD_blank_)

The proliferation rate = (OD_nsPEFs_—OD_control_)/(OD_control_—OD_blank_)

### 2.4 Measurement of intracellular free Ca^2+^

The concentration of intracellular Ca^2+^ concentration was measured using the fluorescence Ca^2+^ indicator Fluo-4 AM according to a previous report[19]. When cells began to grow exponentially, they were treated with 10 pulses of nsPEFs with 100 ns durations at 5 kV/cm, 10 kV/cm, 20 kV/cm electric fields. After nsPEFs treatment, the cells was washed with Hank's Balanced Salt Solution (HBSS) twice, and loaded with 4 μM Fluo-4 AM (Beyotime Institute of Biotechnology, Jiangsu, China) for 45 min in the dark. Then the cells were rinsed with HBSS twice and incubated for another 20 min at 37°C to ensure that Fluo-4 AM had completely transformed into Fluo-4 in the cells. Image were taken with a laser scanning confocal microscope. Detection of intracellular Ca^2+^ was carried out by a BD LSRFortessa SORP cytometer (BD Bioscience). The Ca^2+^ concentration was expressed as the mean fluorescence intensity of each treated group compared with that of the vehicle control group.

### 2.5 Measurement of intracellular reactive oxygen species

Intracellular ROS levels were detected using the reactive oxygen species assay kit (Beyotime Institute of Biotechnology, Jiangsu, China) according to the manufacturer’s protocol. PIEC cells were incubated with 10 μM dichlorofluorescein diacetate (DCFH-DA) for 20 minutes at 37°C. After washing twice, cells were exposed to nsPEFs. Fluorescence intensity was measured by a flow cytometer.

### 2.6 Measurement of Nitric oxide

For the measurement of nitric oxide, normal saline (0.90% w/v of NaCl) was used as the experimental solution for studying exogenous NO production and PIEC cells were suspended in normal saline. After nsPEFs treatment, the solution was centrifuged for 3 min to remove PIEC cells. Then, the total NO concentration stimulated by nsPEFs in normal saline was detected by measuring the concentration of nitrate and nitrite, a stable metabolite of NO by modified Griess reaction method. Total Nitric oxide assay kit (Beyotime Biotechnology China) was used. Experiments were repeated three times.

### 2.7 Statistics

The statistical significance was calculated by ANOVA analysis in SPSS version 22.0. An analysis of variance (ANOVA) was conducted to compare the effects of different nsPEFs treatments on PIEC cells, and significant differences between the mean values were identified by the Student-Newman-Keul’s multiple range test with a confidence level at P < 0.05.

## Result

### 3.1 Cell viability

**Fig 3:**
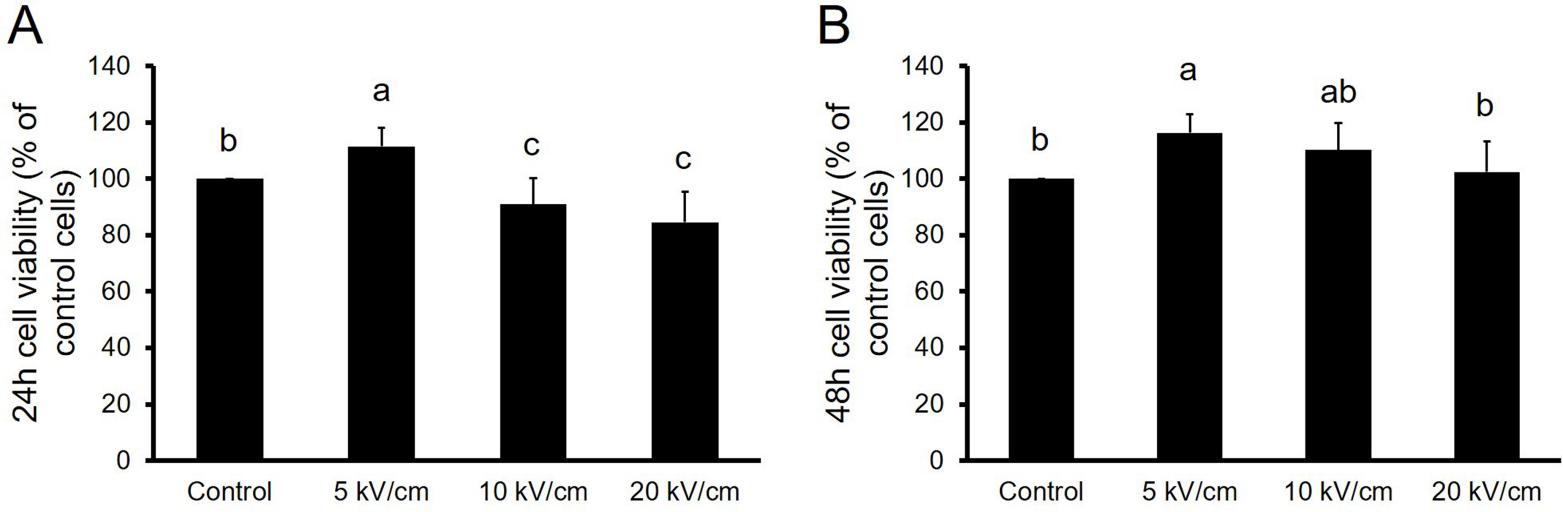
Viability of PIEC cells exposed to 10 pulse of nsPEFs measured by CCK-8 assay after incubation for 24h, 48h. Bar labeled with different lowercase letters indicate a significant difference according to the Student-Newman-Keul’s multiple range test (P<0.05). Date were expressed as mean ± SD (n = 9).

In order to investigate the proliferative effect of nsPEFs on PIEC, cell survival for different exposure parameters was determined by CCK-8 assay after the application of 10 high voltage pulses with field strengths of 5, 10 and 20 kV/cm. Fig 3 depicts the proliferative effect of nsPEFs on PIEC cells. As shown in Fig 3A, when a low field strength (5 kV/cm, 10 pulses) was applied, PIEC cell viability (111.45±8.46%) was increased by 11% of the control group at 24 h and the proliferative rate was significantly (p<0.05) increased compared with the control group. However, after 10 kV/cm and 20 kV/cm, 10 pulses treatments, the growth survival rates reflected to be 90.91±10.96%, 84.60±11.42%. Consequently, high field strengths (over 10 kV/cm) showed an inhibitory effect on PIEC cells. Additionally, Fig 3B reflects the proliferative effect of the PIEC cells after incubation for 48 hours. The growth survival rate was a significant 116.26±9.24% under the low field strength (5 kV/cm). Interestingly, the inhibitory effect of nsPEFs on PIEC cells disappeared under high field strengths (over 10 kV/cm) and the response of PIEC cells to nsPEFs was different under a dose (different pulse parameter and incubation time) dependent manner.

### 3.2 Effect of nsPEFs on intracellular Ca^2+^

**Fig 4:**
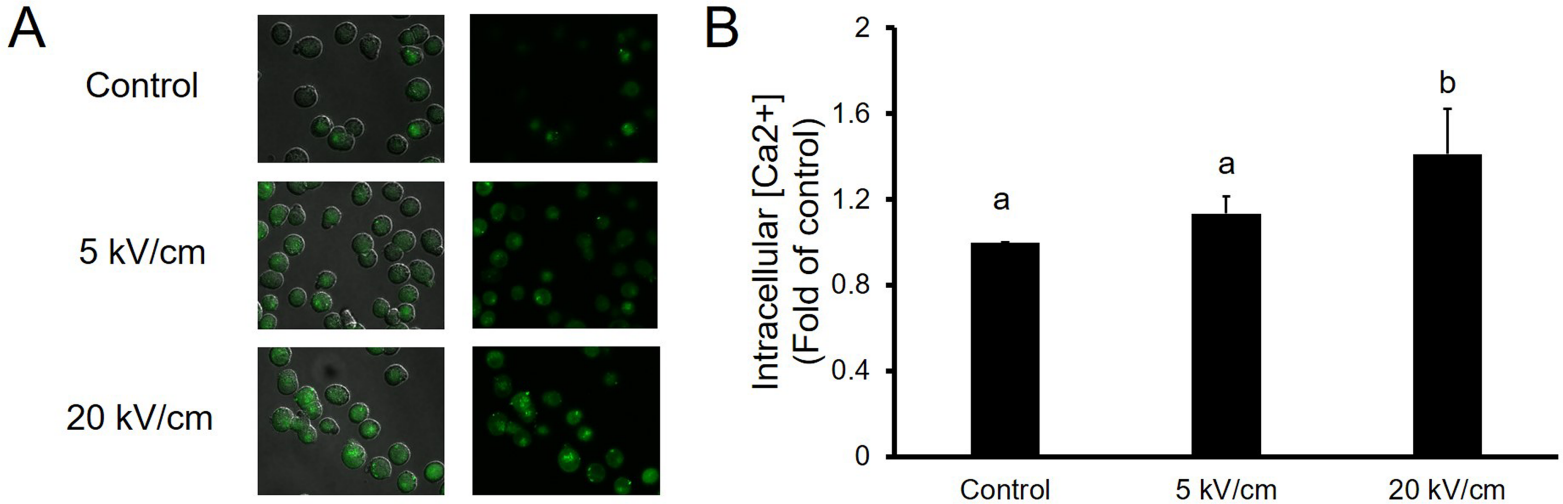
Effect of nsPEFs on intracellular Ca2+ concentrations in PIEC cells. (A) Representative fluorescence image in PIEC cells after nsPEFs treatment. (B) Bar diagram showing the quantitative date of PIEC cells after nsPEFs treatment. Bar labeled with different lowercase letters indicate a significant difference according to the Student-Newman-Keul’s multiple range test (P<0.05). Bars indicate average expression (±SD) of three replicates.

Intracellular calcium level caused by the entry of external calcium and its release from internal stores was found to be related to cell proliferation[20]. In order to verify that the proliferative effect of nsPEFs was caused by the intracellular calcium level, we measured the intracellular calcium concentration utilizing the Fluo-4 fluorescence indicator after nsPEFs treatment with 10 pulses of nsPEFs with 100 ns durations at 5 kV/cm, 20 kV/cm electric fields. The effect of nsPEFs on the intracellular calcium level is visualized in Fig 4 and the results indicate that nsPEFs made the green fluorescence intensity higher than control group. This reflects that the intracellular Ca^2+^ concentration of PIEC cells was significantly increased after nsPEFs treatment. After the application of 10 high voltage pulses with field strengths of 5 and 20 kV/cm, the relative fluorescence intensity of reactive oxygen species were 1.13-fold, and 1.41-fold (p<0.05) compared with the control group. It is also notable that calcium levels in the nsPEFs-exposed cells were dependent on the electric field strength of nsPEFs pulses applied.

### 3.3 Reactive oxygen species induced by nsPEFs

**Fig 5:**
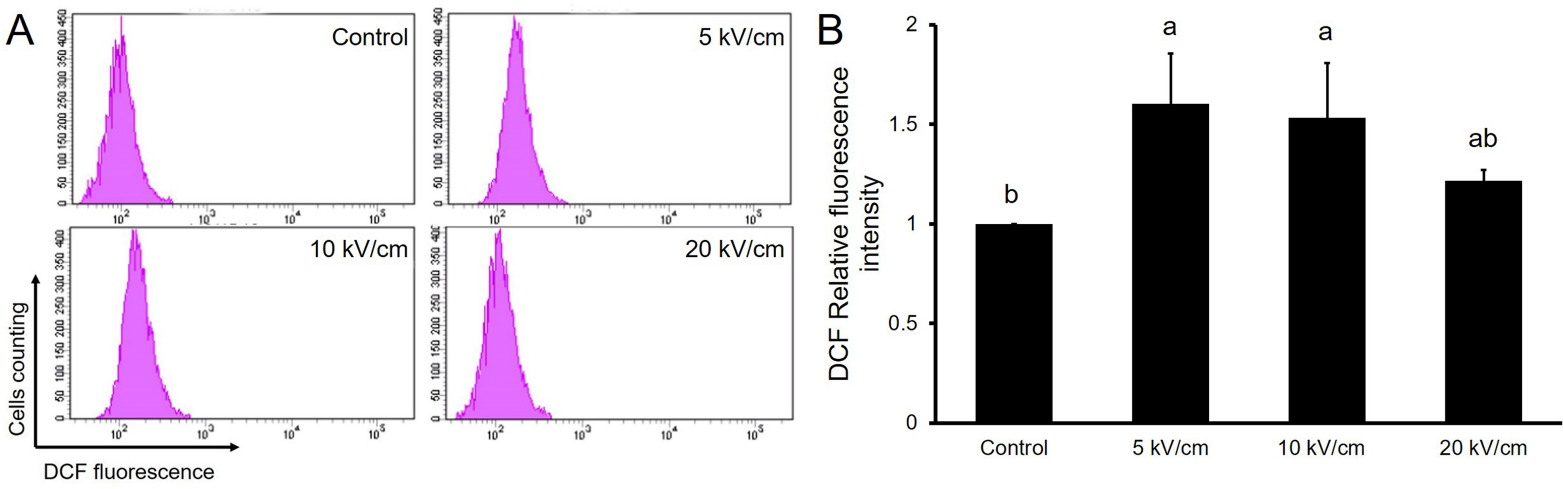
Intracellular reactive oxygen species of PIEC cells after application of 10 high voltage pulses with field strengths of 5, 10 and 20 kV/cm were measured using a flow cytometry. Bar labeled with different lowercase letters indicate a significant difference according to the Student-Newman-Keul’s multiple range test (P<0.05). Bars indicate average expression (±SD) of three replicates.

ROS act as second messengers and can influence a variety of cellular processes including growth factor responses and cell survival[21]. To determine if nsPEFs increased ROS, we measured the intracellular ROS of PIEC cells after nsPEFs treatment. As shown in Fig 5, the intracellular ROS concentration of PIEC cells increased after being exposed to nsPEFs. After the application of 10 high voltage pulses with field strengths of 5, 10 and 20 kV/cm, the relative fluorescence intensity of reactive oxygen species were 1.60-fold (p<0.05), 1.53-fold (p<0.05) and 1.21-fold (p<0.05) compared with the control group, respectively. More interestingly, the concentration of intracellular ROS of cells treated with nsPEFs at 5 kV/cm was higher than that treated with nsPEFs at 20 kV/cm. Such results suggest that nsPEFs promote the proliferation of PIEC cells in a ROS depended manner.

### 3.4 NsPEFs induced increase in extracellular NO production

**Fig 6:**
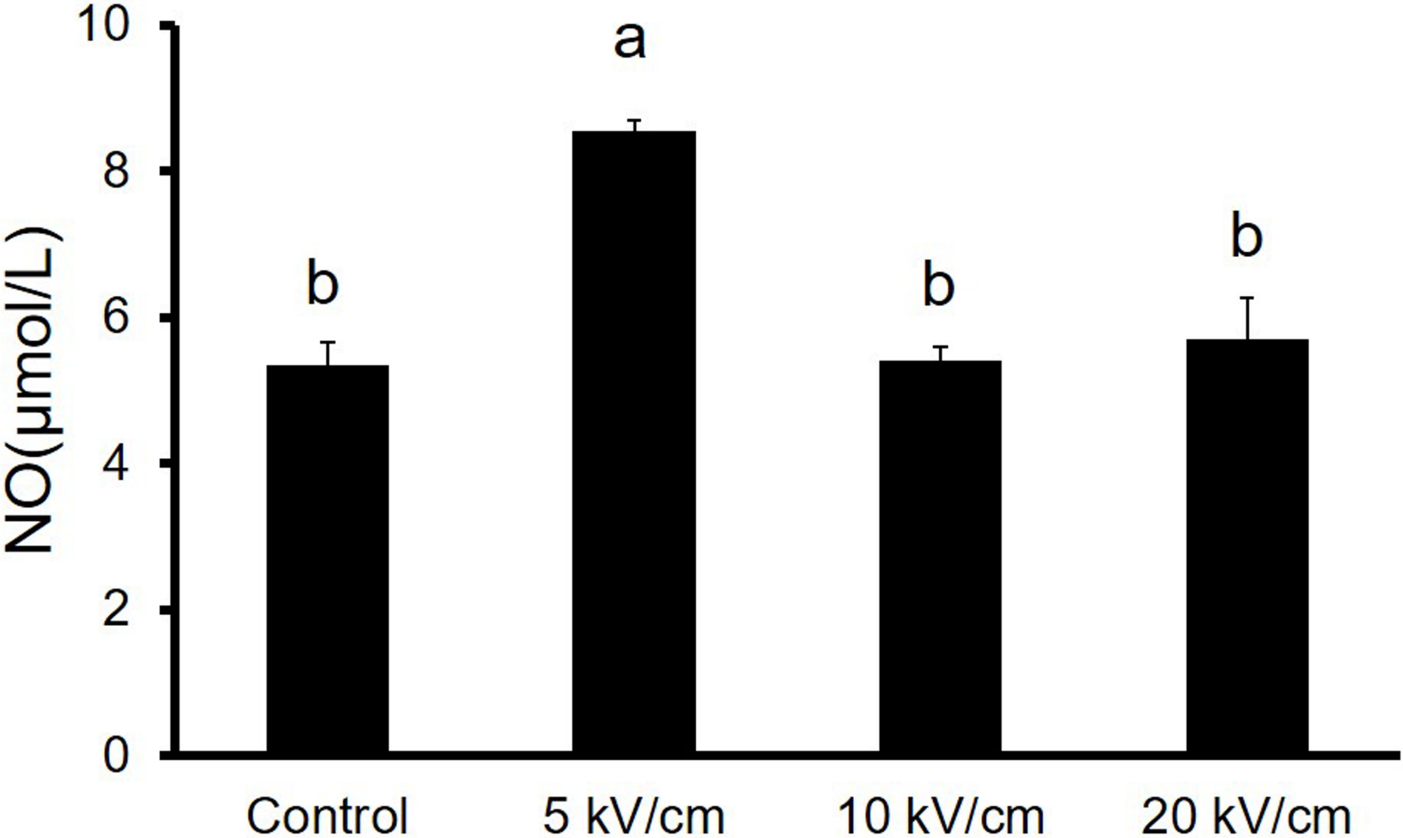
The effect of nsPEFs on extracellular NO production in the nsPEFs treated buffer system (with PIEC cells). Bar labeled with different lowercase letters indicate a significant difference according to the Student-Newman-Keul’s multiple range test (P<0.05). Bars indicate average expression (±SD) of three replicates.

As a signaling molecule, NO regulates various physiological and pathophysiological processes, such as vascular functions (angiogenesis, blood flow, vascular permeability)[22]. To determine whether nsPEFs induced proliferation is related to NO, the total NO concentration was detected in this study. We tested the total NO concentration of each sample with PIEC cells as a means to eliminate the effects of the RPMI-1640 medium after different nsPEFs treatment. As depicted in Fig 6, the total NO concentrations in the NaCl solution of the control group was 5.34±0.31μmol/L. Meanwhile, the NO concentration detected in nsPEFs treatment groups were 8.54±0.15, 5.42±0.19, 5.70±0.56μmol/L for 5 to 20 kV/cm, 10 pulses and the total NO concentration in the NaCl solution treated with nsPEFs at 5 kV/cm was significantly higher than the control group. These results suggest that NO may be a factor in promoting the proliferation of PIEC cells by nsPEFs treatment.

## Discussion

Nanosecond pulsed electric fields (nsPEFs) with short pulse duration, low energy density and non-thermal effects possess various practical applications, and is most often applied for cell fusion, separation of nucleic acids and proteins, as well as in electroporation for molecular transport [23, 24]. It is well-known that nsPEFs can be used as a novel cancer therapy if high electric field (over 20 kV/cm) is involved [25]. In this study, we discovered that nanosecond pulsed electric fields have a dose-dependent effect on cells. Specifically speaking, electric pulses with high electric field intensity can inhibit cell growth, while nsPEFs can induce proliferative effect under low electric field strength. Zhang et al. demonstrated that exposing chondrocytes to nsPEFs at low electric field strength led to enhanced proliferation and dedifferentiation[15]. Additional, Guo et al. also determined that 20 pulses of nsPEFs at 15 kV/cm increased avermectin production by 42% and reduced the time for reaching a plateau in the fermentation process[16]. Both academic analyses and medical experiment results indicated that a low field intensity was able to induce cell proliferation. Base on this consideration, we investigated the proliferative effect of PIEC cells after being exposed to nsPEFs at a low electric field strength. Consequently, our study showed that exposures to nanosecond pulsed electric fields were able to temporarily change the viability of PIEC cells. Furthermore, we also found that low field strengths (5 kV/cm) reflected a significant proliferative effect (P<0.05) on PIEC cells and the proliferative effect had a time-dependent manner. Meanwhile, high field strengths (over 10 kV/cm) showed an inhibitory effect on PIEC cells. These results are in accordance with the previous studies which indicated that nsPEFs treatment with low intensity could induce cell proliferation of S.avermitilis, while high field intensity would lead to an obvious inhibition effect[16]. Our results indicated that nsPEFs at low electric field can promote the proliferation of PIEC cells, which had a potential to stimulate endothelial cells to accelerate stent endothelialization.

We examined the intracellular free calcium levels after exposure to nsPEFs and found that intracellular free calcium concentrations increased after nsPEFs treatment compared to the control group. Previous studies [26, 27] have determined that the nsPEFs can cause a large number of nanopores on both the plasma membrane and intracellular membrane, and concluded that the intracellular membrane is more susceptible to nsPEFs than plasma membranes. It is notable that those nanopores can induce a variety of responses in cells such an increase in the intracellular free calcium level[28]. Scarlett[26] et al discovered that the [Ca^2+^]_i_ response was due to the release of Ca^2+^ from the intracellular store at a low electric field strength and that the increase in [Ca^2+^]_i_ was caused by both an internal release and an influx across the plasma membrane at a high electric field strength. Our results aligned with previous theoretical and experimental studies. At a low field strength of 5 kV / cm, the pulse only acts on the intracellular endoplasmic reticulum, so that the endoplasmic reticulum calcium library was released. At a high field strength of 20 kV / cm, the pulse not only leads to the release of calcium in the endoplasmic reticulum, but also causes cell membrane damage, leading to calcium influx in the culture medium. The calcium signal created by the entry of external calcium and its release from internal stores related to the cell proliferation by activating the immediate early genes responsible for inducing resting cells (G0) to re-enter the cell cycle [20]. When a low field strength of 5 kV/cm and 10 pulses were applied, PIEC cell viability was increased due to the increase of intracellular free calcium concentration. Meanwhile, high field strengths (over 10 kV/cm) reflected an inhibitory effect on PIEC cells due to the damage of plasma membranes. Consequently, such results indicated that changes of intracellular calcium level may be a factor for enhancing the proliferation in PIEC cell by nsPEFs treatment.

Reactive oxygen species such as superoxide anion (O_2_^-^), hydrogen peroxide (H_2_O_2_), and hydroxyl radical (HO•), consist of radical and non-radical oxygen species formed by the partial reduction of oxygen. As “two-faced” molecules, ROS are involved in various complex signaling pathways and are critical to the fate of both healthy and diseased cells. Additionally, a low level of ROS can directly interact with critical signing molecular as a means to initiate signaling in a broad variety of cellular processes, such as proliferation and survival. Meanwhile, higher levels of ROS cause disruptions of cellular processes through the non-specific attack of proteins, lipids, and DNA, which can cause cell dysfunction [21, 29].We measured the intracellular ROS of PIEC cells after nsPEFs treatment and found that nsPEFs were able to significantly induced an increase in intracellular ROS levels (p<0.05) compared with the control group. This result is consistent with the previous studies which reflected that nsPEFs could lead to elevated intracellular ROS levels [30, 31]. Nuccitelli[30] et al. demonstrated that 100ns, 30 kV/cm nsPEFs can stimulate an increase in ROS proportional to the pulse number. NsPEFs can induce a small increase in the intracellular ROS level that activates signaling pathway to enhance cell proliferation through the use of low-power pulses. This result indicated that nsPEFs promoted the proliferation of PIEC cells in a ROS depended manner.

Previous studies[14, 25] reported that nsPEFs can induce exogenous NO and endogenous NO. Guo[25] et al. determined that the total NO concentration in the buffer solution of controls was significantly lower than those of nsPEFs treatment groups. Furthermore, as a signaling molecule, NO is not only able to expand the blood vessels, but can also spread to the vascular lumen, inhibition of platelet aggregation and adhesion, which prevent neutrophils and monocytes from adhering to the blood vessel wall, creating an anti-thrombotic performance on the endothelial surface [22, 32, 33]. The therapeutic and biosafety effects of NO highly depend on its concentration and location in the body. Lower concentrations of NO have been suggested to exert a direct effect on processes such as cell proliferation and survival; whereas higher concentrations have an indirect effect through both oxidative and nitrosative stresses. It is also notable that it can inhibit the proliferation of smooth muscle cells[34]. In order to determine whether nsPEFs induce cell proliferation is related to NO, we detected the change of NO concentration in an NaCl solution. The results reflected that NO experienced an obvious increase (P<0.05) after treatment with nsPEFs at 5 kV/cm compared with the control group. The existence of exogenous NO in the NaCl solution may be dependent on the arc discharge during the pulse electric field[35]. According to the arc discharge theory, the nitrogen and oxygen dissolved in the NaCl solution could be dissociated into active species in the pulsed high voltage discharge system. These results suggested that NO could be a factor used in enhancing proliferation in PIEC cells through nsPEFs treatment.

As a novel technology, nsPEFs has received great attention in practical applications of medicine and biology. Furthermore, studies [36, 37] have shown that nsPEFs may have a selective effect on different cells, which indicates that different cells have various types of proliferation effects after same pulse parameters treatment. In the treatment of ISR, we are interested in combining performances, since it is able to inhibit the proliferation of smooth muscle cell while also promoting the proliferation of endothelial cells. NsPEFs should be useful for further theoretic and experimental studies in this disease treatment. In the further studies, we will explore the growth of endothelial cells on the stent in vivo under the stimulation of nanosecond pulsed electric fields.

## Conclusion

To Summarize our data, appropriate pulse parameters are able to enhance cell proliferation on PIEC cells which may involve the intracellular Ca^2+^ concertation, as well as ROS and NO production in nsPEFs treatment system. Our research provides a new method in promoting the cell proliferation of PIEC cells. These results suggest that nsPEFs may have a potential application in the process of accelerating the stent endothelialization.

## Supporting information

S1 Fig The absorbance of PIEC cells measured by CCK-8 assay after nsPEFs treatment immediately. (TIF)

S2 Fig The Representative microscopical image of PIEC cells after nsPEFs treatment at 24h (scale, bar 100μm). (TIF)

## References

[1] Roger VL, Go AS, Lloyd-Jones DM, Benjamin EJ, Berry JD, Borden WB, et al Executive Summary: Heart Disease and Stroke Statistics-2012 Update A Report From the American Heart Association. Circulation. 2012;125(1):188–97. PubMed PMID: WOS:000299169100035. doi: 10.1161/CIR.0b013e3182456d46 22215894 10.1161/CIR.0b013e3182456d46

[2] Mani G, Feldman MD, Patel D, Agrawal CM. Coronary stents: a materials perspective. Biomaterials. 2007;28(9):1689–710. doi: 10.1016/j.biomaterials.2006.11.042 . 17188349 10.1016/j.biomaterials.2006.11.042

[3] Stone GW, Ellis SG, Cox DA, Hermiller J, O'Shaughnessy C, Mann JT, et al A polymer-based, paclitaxel-eluting stent in patients with coronary artery disease. New Engl J Med. 2004;350(3):221–31. PubMed PMID: WOS:000188078400005. doi: 10.1056/NEJMoa032441 14724301 10.1056/NEJMoa032441

[4] Morice M, Serruys PW, Sousa JE, Fajadet J, Hayashi EB, Perin M, et al A randomized comparison of a sirolimus-eluting stent with a standard stent for coronary revascularization. New Engl J Med. 2002;346(23):1773–80. PubMed PMID: WOS:000176012500002. doi: 10.1056/NEJMoa012843 12050336 10.1056/NEJMoa012843

[5] Takahashi S, Kaneda H, Tanaka S, Miyashita Y, Shiono T, Taketani Y, et al Late angiographic stent thrombosis after sirolimus-eluting stent implantation. Circ J. 2007;71(2):226–8. PubMed PMID: WOS:000243861000011. 17251672 10.1253/circj.71.226

[6] Avci-Adali M, Ziemer G, Wendel HP. Induction of EPC homing on biofunctionalized vascular grafts for rapid in vivo self-endothelialization—a review of current strategies. Biotechnol Adv. 2010;28(1):119–29. doi: 10.1016/j.biotechadv.2009.10.005 . 19879347 10.1016/j.biotechadv.2009.10.005

[7] Wu X, Zhao Y, Tang C, Yin T, Du R, Tian J, et al Re-Endothelialization Study on Endovascular Stents Seeded by Endothelial Cells through Up- or Downregulation of VEGF. ACS Appl Mater Interfaces. 2016;8(11):7578–89. doi: 10.1021/acsami.6b00152 . 26925508 10.1021/acsami.6b00152

[8] Shirota T, Yasui H, Shimokawa H, Matsuda T. Fabrication of endothelial progenitor cell (EPC)-seeded intravascular stent devices and in vitro endothelialization on hybrid vascular tissue. Biomaterials. 2003;24(13):2295–302. PubMed PMID: WOS:000182280400019. 12699666 10.1016/s0142-9612(03)00042-5

[9] Aoki J, Serruys PW, van Beusekom H, Ong ATL, McFadden EP, Sianos G, et al Endothelial progenitor cell capture by stents coated with antibody against CD34—The HEALING-FIM (healthy endothelial accelerated lining inhibits neointimal growth-first in man) registry. J Am Coll Cardiol. 2005;45(10):1574–9. PubMed PMID: WOS:000229121000003. doi: 10.1016/j.jacc.2005.01.048 15893169 10.1016/j.jacc.2005.01.048

[10] Schoenbach KH, Beebe SJ, Buescher ES. Intracellular effect of ultrashort electrical pulses. Bioelectromagnetics. 2001;22(6):440–8. PubMed PMID: WOS:000170747200007. 11536285 10.1002/bem.71

[11] Wu S, Wang Y, Guo J, Chen Q, Zhang J, Fang J. Nanosecond pulsed electric fields as a novel drug free therapy for breast cancer: an in vivo study. Cancer Lett. 2014;343(2):268–74. doi: 10.1016/j.canlet.2013.09.032 . 24099912 10.1016/j.canlet.2013.09.032

[12] Kulbacka J. Nanosecond pulsed electric fields (nsPEFs) impact and enhanced Photofrin II((R)) delivery in photodynamic reaction in cancer and normal cells. Photodiagnosis Photodyn Ther. 2015;12(4):621–9. doi: 10.1016/j.pdpdt.2015.11.002 . 26563460 10.1016/j.pdpdt.2015.11.002

[13] Hargrave B, Li F. Nanosecond Pulse Electric Field Activated-Platelet Rich Plasma Enhances the Return of Blood Flow to Large and Ischemic Wounds in a Rabbit Model. Physiol Rep. 2015;3(7). doi: 10.14814/phy2.12461 ; PubMed Central PMCID: PMCPMC4552537. 26197934 10.14814/phy2.12461 PMC4552537

[14] Su B, Guo J, Nian W, Feng H, Wang K, Zhang J, et al Early Growth Effects of Nanosecond Pulsed Electric Field (nsPEFs) Exposure onHaloxylon ammodendron. Plasma Processes and Polymers. 2015;12(4):372–9. doi: 10.1002/ppap.201400131

[15] Zhang K, Guo J, Ge Z, Zhang J. Nanosecond pulsed electric fields (nsPEFs) regulate phenotypes of chondrocytes through Wnt/beta-catenin signaling pathway. Sci Rep. 2014;4:5836 doi: 10.1038/srep05836 . 25060711 10.1038/srep05836 PMC5376156

[16] Guo J, Ma R, Su B, Li Y, Zhang J, Fang J. Raising the avermectins production in Streptomyces avermitilis by utilizing nanosecond pulsed electric fields (nsPEFs). Sci Rep. 2016;6:25949 doi: 10.1038/srep25949 ; PubMed Central PMCID: PMCPMC4867605. 27181521 10.1038/srep25949 PMC4867605

[17] Choi DH, Kang SN, Kim SM, Gobaa S, Park BJ, Kim IH, et al Growth factors-loaded stents modified with hyaluronic acid and heparin for induction of rapid and tight re-endothelialization. Colloids Surf B Biointerfaces. 2016;141:602–10. doi: 10.1016/j.colsurfb.2016.01.028 . 26928466 10.1016/j.colsurfb.2016.01.028

[18] Kolb JF, Kono S, Schoenbach KH. Nanosecond pulsed electric field generators for the study of subcellular effects. Bioelectromagnetics. 2006;27(3):172–87. PubMed PMID: WOS:000236411600002. doi: 10.1002/bem.20185 16304697 10.1002/bem.20185

[19] Luo DL, Yang DM, Lan XM, Li KT, Li XD, Chen J, et al Nuclear Ca2+ sparks and waves mediated by inositol 1,4,5-trisphosphate receptors in neonatal rat cardiomyocytes. Cell Calcium. 2008;43(2):165–74. PubMed PMID: WOS:000254158600006. doi: 10.1016/j.ceca.2007.04.017 17583790 10.1016/j.ceca.2007.04.017 PMC2266086

[20] Berridge MJ. Calcium Signaling and Cell-Proliferation. Bioessays. 1995;17(6):491–500. doi: 10.1002/bies.950170605 PubMed PMID: WOS:A1995RE63600004. 7575490 10.1002/bies.950170605

[21] Schieber M, Chandel NS. ROS Function in Redox Signaling and Oxidative Stress. Curr Biol. 2014;24(10):R453–R62. PubMed PMID: WOS:000336340000021. doi: 10.1016/j.cub.2014.03.034 24845678 10.1016/j.cub.2014.03.034 PMC4055301

[22] Tousoulis D, Kampoli AM, Papageorgiou CTN, Stefanadis C. The Role of Nitric Oxide on Endothelial Function. Curr Vasc Pharmacol. 2012;10(1):4–18. PubMed PMID: WOS:000300289200003. 22112350 10.2174/157016112798829760

[23] Steuer A, Schmidt A, Laboha P, Babica P, Kolb JF. Transient suppression of gap junctional intercellular communication after exposure to 100-nanosecond pulsed electric fields. Bioelectrochemistry. 2016;112:33–46. doi: 10.1016/j.bioelechem.2016.07.003 . 27439151 10.1016/j.bioelechem.2016.07.003

[24] Chen X, Yin S, Hu C, Chen X, Jiang K, Ye S, et al Comparative study of nanosecond electric fields in vitro and in vivo on hepatocellular carcinoma indicate macrophage infiltration contribute to tumor ablation in vivo. PLoS One. 2014;9(1):e86421 doi: 10.1371/journal.pone.0086421 ; PubMed Central PMCID: PMCPMC3903538. 24475118 10.1371/journal.pone.0086421 PMC3903538

[25] Guo J, Wang Y, Wang J, Zhang J, Fang J. Radiosensitization of oral tongue squamous cell carcinoma by nanosecond pulsed electric fields (nsPEFs). Bioelectrochemistry. 2017;113:35–41. doi: 10.1016/j.bioelechem.2016.09.002 . 27676191 10.1016/j.bioelechem.2016.09.002

[26] Scarlett SS, White JA, Blackmore PF, Schoenbach KH, Kolb JF. Regulation of intracellular calcium concentration by nanosecond pulsed electric fields. Biochim Biophys Acta. 2009;1788(5):1168–75. doi: 10.1016/j.bbamem.2009.02.006 . 19230822 10.1016/j.bbamem.2009.02.006

[27] Pakhomov AG, Gianulis E, Vernier PT, Semenov I, Xiao S, Pakhomova ON. Multiple nanosecond electric pulses increase the number but not the size of long-lived nanopores in the cell membrane. Bba-Biomembranes. 2015;1848(4):958–66. PubMed PMID: WOS:000350518800008. doi: 10.1016/j.bbamem.2014.12.026 25585279 10.1016/j.bbamem.2014.12.026 PMC4331219

[28] Beier HT, Roth CC, Tolstykh GP, Ibey BL. Resolving the spatial kinetics of electric pulse-induced ion release. Biochem Bioph Res Co. 2012;423(4):863–6. PubMed PMID: WOS:000307032400042. 10.1016/j.bbrc.2012.06.055 22713455

[29] Ray PD, Huang BW, Tsuji Y. Reactive oxygen species (ROS) homeostasis and redox regulation in cellular signaling. Cell Signal. 2012;24(5):981–90. PubMed PMID: WOS:000301686600002. doi: 10.1016/j.cellsig.2012.01.008 22286106 10.1016/j.cellsig.2012.01.008 PMC3454471

[30] Nuccitelli R, Lui K, Kreis M, Athos B, Nuccitelli P. Nanosecond pulsed electric field stimulation of reactive oxygen species in human pancreatic cancer cells is Ca(2+)-dependent. Biochem Biophys Res Commun. 2013;435(4):580–5. doi: 10.1016/j.bbrc.2013.05.014 ; PubMed Central PMCID: PMCPMC3730523. 23680664 10.1016/j.bbrc.2013.05.014 PMC3730523

[31] Pakhomova ON, Khorokhorina VA, Bowman AM, Rodaite-Riseviciene R, Saulis G, Xiao S, et al Oxidative effects of nanosecond pulsed electric field exposure in cells and cell-free media. Arch Biochem Biophys. 2012;527(1):55–64. doi: 10.1016/j.abb.2012.08.004 ; PubMed Central PMCID: PMCPMC3459148. 22910297 10.1016/j.abb.2012.08.004 PMC3459148

[32] Fukumura D, Kashiwagi S, Jain RK. The role of nitric oxide in tumour progression. Nat Rev Cancer. 2006;6(7):521–34. doi: 10.1038/nrc1910 . 16794635 10.1038/nrc1910

[33] Napoli C, Paolisso G, Casamassimi A, Al-Omran M, Barbieri M, Sommese L, et al Effects of Nitric Oxide on Cell Proliferation Novel Insights. J Am Coll Cardiol. 2013;62(2):89–95. PubMed PMID: WOS:000321338600002. doi: 10.1016/j.jacc.2013.03.070 23665095 10.1016/j.jacc.2013.03.070

[34] Li HG, Forstermann U. Prevention of Atherosclerosis by Interference with the Vascular Nitric Oxide System. Curr Pharm Design. 2009;15(27):3133–45. PubMed PMID: WOS:000269149100005. 10.2174/138161209789058002 19754387

[35] Bian W, Song X, Liu D, Zhang J, Chen X. Actions of nitrogen plasma in the 4-chrolophenol degradation by pulsed high-voltage discharge with bubbling gas. Chemical Engineering Journal. 2013;219:385–94. doi: 10.1016/j.cej.2012.12.074

[36] Orban C, Perez-Garcia E, Bajnok A, McBean G, Toldi G, Blanco-Fernandez A. Real Time Kinetic Flow Cytometry Measurements of Cellular Parameter Changes Evoked by Nanosecond Pulsed Electric Field. Cytom Part A. 2016;89a(5):472–9. PubMed PMID: WOS:000379604000008. 10.1002/cyto.a.22838 26990601

[37] Yao CG, Hu XQ, Mi Y, Li CX, Sun CX. Window Effect of Pulsed Electric Field on Biological Cells. Ieee T Dielect El In. 2009;16(5):1259–66. PubMed PMID: WOS:000271022800006.

