# Supplementary figures and images for: Nanosecond pulsed electric fields promoting the proliferation of porcine iliac endothelial cells: An in vitro study
Source: PLoS One. 2018 May 1;13(5):e0196688. doi: 10.1371/journal.pone.0196688 (PMC5929542; doi:10.1371/journal.pone.0196688)

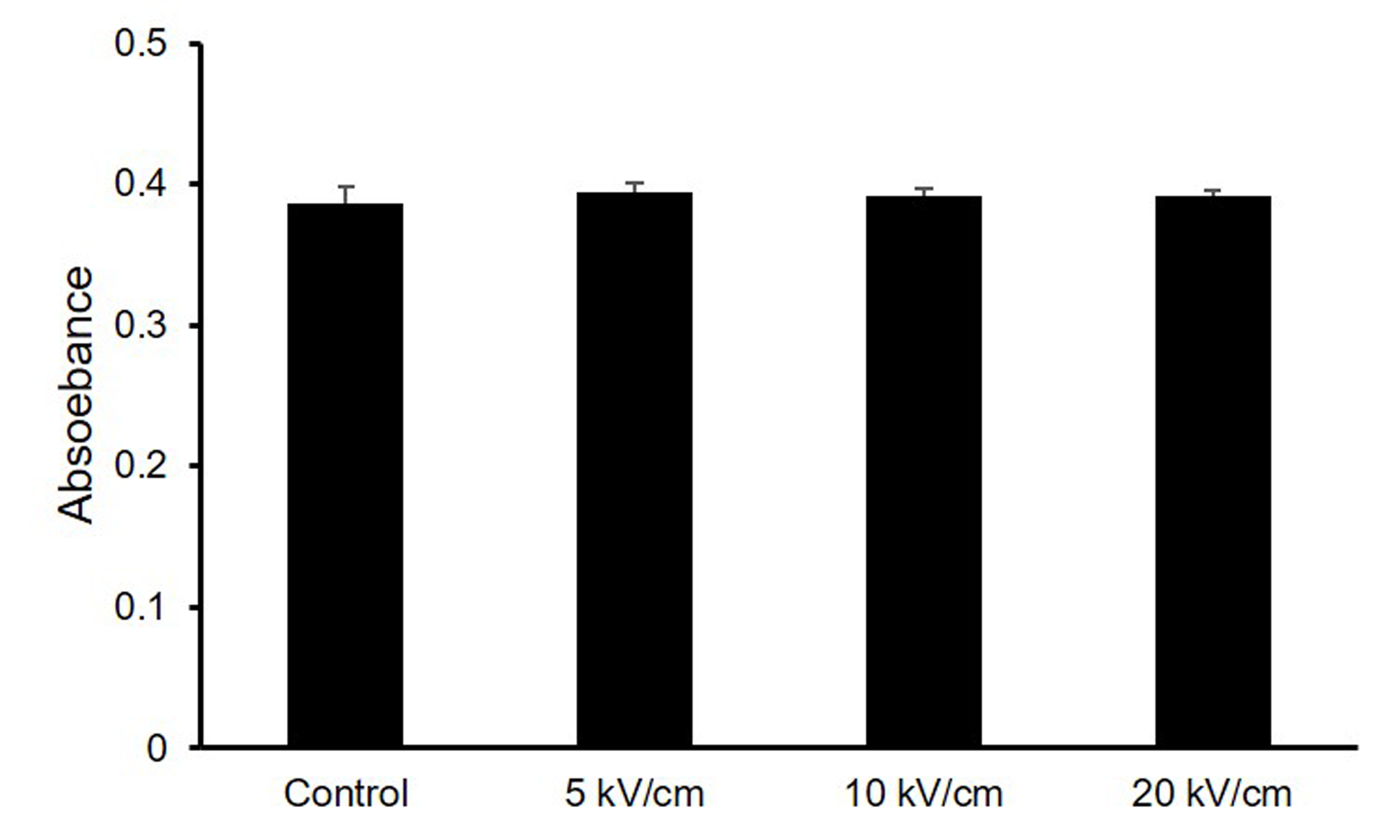

Supplement: S1 Fig — (TIF) [file pone.0196688.s001.tif]

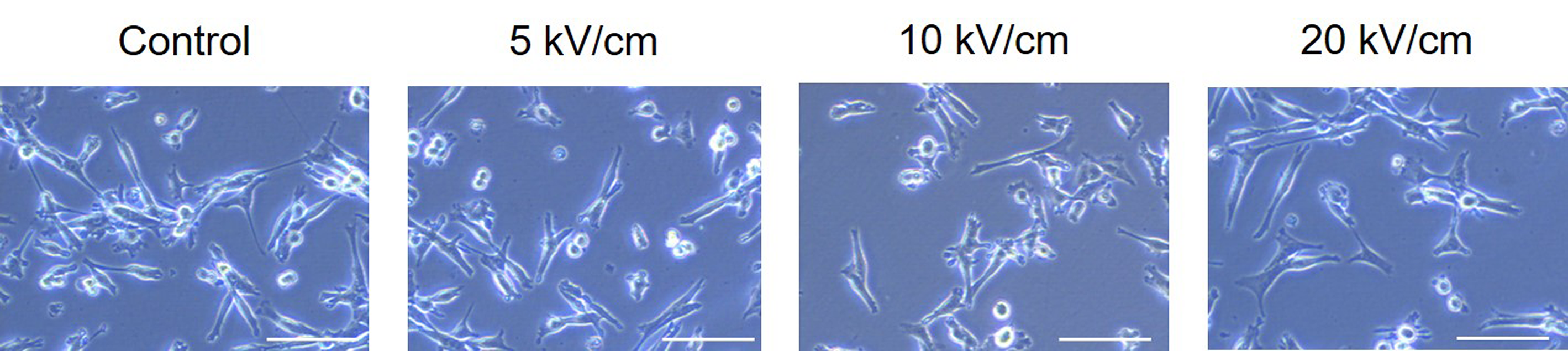

Supplement: S2 Fig — (TIF) [file pone.0196688.s002.tif]
